# Aufwachsen in einem psychisch belasteten Familienumfeld: Ergebnisse der bundesweit repräsentativen Studie „Kinder in Deutschland 0–3 2022“

**DOI:** 10.1007/s00103-024-03979-2

**Published:** 2024-11-13

**Authors:** Maria Hänelt, Anna Neumann, Ulrike Lux, Ilona Renner

**Affiliations:** 1 Nationales Zentrum Frühe Hilfen, Bundeszentrale für gesundheitliche Aufklärung, Maarweg 149–161, 50825 Köln, Deutschland; 2 Nationales Zentrum Frühe Hilfen, Deutsches Jugendinstitut e. V., München, Deutschland

**Keywords:** Frühe Hilfen, Psychische Gesundheit, Kindergesundheit, Erziehungskompetenz, Regulationsstörung, Early childhood intervention, Parental mental health, Child health, Parenting, Regulatory disorders

## Abstract

**Hintergrund:**

Ein psychisch belastetes Familienumfeld kann ein Risiko für die gesunde und altersgerechte Entwicklung von Säuglingen und Kleinkindern darstellen. Ungeklärt ist, wie weit psychische Belastungen aktuell in der Gesamtpopulation von Eltern mit kleinen Kindern verbreitet sind und ob der Anteil psychisch belasteter Eltern in den zurückliegenden Jahren multipler gesellschaftlicher Krisen angestiegen ist. Zudem ist unklar, wie sich Zusammenhänge zwischen der psychischen Belastung der Eltern, deren Erziehungskompetenzen sowie der frühkindlichen Entwicklung aktuell darstellen.

**Methoden:**

In der bundesweiten, repräsentativen Befragung „Kinder in Deutschland – KiD 0–3 2022“ dokumentierten 258 Kinderärztinnen und -ärzte im Rahmen einer Vorsorgeuntersuchung die Entwicklung von 7818 Säuglingen und Kleinkindern. Die Eltern der Kinder beantworteten u. a. Fragen zu ihren psychischen Belastungen (Angst und Depressivität) und ihren Erziehungskompetenzen. Die Prävalenzen von 2022 wurden mit Ergebnissen der Vorgängerstudie aus dem Jahr 2015 verglichen.

**Ergebnisse:**

2022 berichtete etwa ein Fünftel aller Eltern (21,5 %) eine moderate bis klinisch bedeutsame psychische Belastung (2015: 15,7 %). Psychisch belastete Eltern äußerten signifikant häufiger Zweifel an der eigenen elterlichen Kompetenz als Eltern ohne psychische Belastung. Ärztinnen und Ärzte dokumentierten bei Kindern, deren Eltern klinisch bedeutsame psychische Belastungen berichteten, signifikant häufiger Hinweise auf eine Regulationsstörung und eine Entwicklungsverzögerung.

**Diskussion:**

Die Ergebnisse verdeutlichen die in den letzten Jahren zunehmende Relevanz psychischer Belastungen bei Eltern mit kleinen Kindern. Der Befund, dass psychische Belastungen der Eltern mit kindlichen Entwicklungsstörungen einhergehen, verdeutlicht einmal mehr die Notwendigkeit, Familien von Beginn an gezielt zu unterstützen.

## Abstract (en)

**Background:**

A mentally stressed family environment can pose a risk to the healthy development of infants and young children. It is unclear how widespread psychological distress currently is in the overall population of parents with young children and whether the proportion of parents with psychological distress has increased in recent years of multiple crises. Furthermore, it is unclear to what extent associations between the psychological distress of parents, their parenting skills, and early childhood development persist when a growing number of parents report psychological distress.

**Methods:**

In a nationwide, representative survey, 258 pediatricians documented the development of 7818 infants and toddlers as part of a preventive medical checkup. The children’s parents answered questions about their mental health (anxiety and depression) and their parenting skills. The 2022 prevalences were compared with the results of the previous study from 2015.

**Results:**

Taken together, around a fifth (21.5%) of all parents reported moderate or clinically significant psychological distress (2015: 15.7%); parents with psychological distress were significantly more likely to express doubts about their own parenting competencies than parents without psychological distress. Pediatricians documented indications of a regulatory disorder and developmental delay significantly more frequently in children whose parents reported clinically significant psychological distress.

**Discussion:**

The results illustrate the increasing relevance of psychological distress among parents with young children in recent years. The finding that parental psychological distress is associated with developmental abnormality in children once again highlights the need to provide families with targeted support from the outset.

## Einleitung

Die psychische Gesundheit der Eltern prägt die Rahmenbedingungen der kindlichen Entwicklung von Beginn an. Für Kinder, die mit einem psychisch erkrankten Elternteil aufwachsen, ist das Risiko erhöht, im Laufe ihres Lebens selbst psychisch zu erkranken [1]. Zusammenhänge zwischen der psychischen Erkrankung eines Elternteils und der kindlichen Entwicklung zeigen sich bereits im Säuglings- und Kleinkindalter. So können sich psychische Belastungen von Eltern kurz- und mittelfristig negativ auf die Regulationsfähigkeiten kleiner Kinder [2, 3] und auf das Erreichen von Meilensteinen einer altersgerechten Entwicklung auswirken [4]. Klinisch bedeutsame Probleme in den Regulationsfähigkeiten (bzw. Regulationsstörungen) des Kindes können sich etwa in einem mehrere Wochen andauernden exzessiven Schreien, Ein- und Durchschlafstörungen oder Fütter- und Gedeihstörungen zeigen [5]. Eine nicht altersgerechte Entwicklung liegt vor, wenn die kindliche Entwicklung von Normwerten abweicht. Insbesondere für eine mütterliche Depression oder Angststörung wurde sowohl in quer- als auch in längsschnittlich angelegten Studien ein Zusammenhang mit solchen kindlichen Entwicklungsrisiken gefunden [6–8].

Der Zusammenhang zwischen einer elterlichen psychischen Erkrankung und einem erhöhten Risiko ungünstiger kindlicher Entwicklungsverläufe kann – neben (epi)genetischen Mechanismen [9] oder Modelllernen [10] – über eine belastete Eltern-Kind-Beziehung vermittelt werden [11]. Es ist empirisch gut belegt, dass sich bspw. eine elterliche Depression negativ auf Erziehungskompetenzen und die Eltern-Kind-Beziehung auswirken kann [8, 12–16]. Depressionen und Angststörungen der Eltern gehen mit unterschiedlichen Symptomen, wie bspw. ständiger Nervosität oder Niedergeschlagenheit, einher, die die Eltern-Kind-Interaktion belasten können [17]. Vor allem negative Kognitionen, die auch eine negative Sicht auf die eigenen Erziehungskompetenzen beinhalten können [4], können zu einer dysfunktionalen Eltern-Kind-Beziehung beitragen [18].

Familien mit kleinen Kindern gehörten zu den Bevölkerungsgruppen, die besonders von den gravierenden Einschränkungen durch Maßnahmen zur Bewältigung der COVID-19-Pandemie betroffen waren [19] und ihre Situation in dieser Zeit als sehr belastend erlebten [20, 21]. Ungeklärt ist, inwieweit die zusätzliche Belastung durch die COVID-19-Pandemie und weitere Krisen, bspw. Kriegsgefahr, Energie- und Klimakrise, mit einer Verschlechterung der psychischen Gesundheit bei Eltern kleiner Kinder einhergeht. Hypothetisiert wird im vorliegenden Beitrag deshalb, dass – verglichen mit der Zeit vor diesen aktuellen Krisen – ein größerer Anteil von Eltern mit kleinen Kindern in Deutschland von psychischen Belastungen betroffen ist. Es wird zudem angenommen, dass die in der oben zitierten Literatur beschriebenen Zusammenhänge zwischen elterlicher psychischer Belastung und kindlichen Entwicklungsrisiken auch unter den aktuellen Bedingungen gelten. Falls beide Annahmen zutreffen, dass sowohl ein Anstieg psychischer Belastungen der Eltern zwischen 2015 und 2022 erfolgte als auch der statistische Zusammenhang zwischen elterlichen psychischen Belastungen und kindlichen Entwicklungsrisiken fortbesteht, könnte dies ein Hinweis darauf sein, dass die Belastungsanzeigen der Eltern nicht ausschließlich Ausdruck einer gestiegenen Sensibilität gegenüber psychischen Belastungen oder einer geringeren Hemmschwelle, diese zu berichten, sind, sondern einen tatsächlichen Anstieg psychischer Belastungen spiegeln, der sich entsprechend auch auf die kindliche Entwicklung auswirkt.

Bisher fehlten für Deutschland aktuelle und bundesweit repräsentative Daten zur psychischen Belastung von Eltern kleiner Kinder sowie zu deren Einschätzung der eigenen Erziehungskompetenzen und der Gesundheit und Entwicklung ihrer Säuglinge und Kleinkinder. Mit der Studie „Kinder in Deutschland – KiD 0–3 2022“, an der sich insgesamt *N* = 7818 Familien mit Kindern bis zu 3 Jahren beteiligten, wird diese Lücke geschlossen [22]. In diesem Artikel sollen anhand eines aktuellen bundesweit repräsentativen Datensatzes zur Situation von Familien mit Säuglingen und Kleinkindern folgende Forschungsfragen empirisch untersucht werden:

1. Wie weit verbreitet sind psychische Belastungen (bzw. Symptome einer Depression oder Angststörung) bei Eltern von kleinen Kindern?
2. Ist der Anteil psychisch belasteter Eltern in den letzten Jahren angestiegen (im Vergleich mit Ergebnissen der Vorgängerstudie KiD 0–3 2015)?
3. Wie unterscheiden sich Eltern mit unterschiedlichen psychischen Belastungsniveaus hinsichtlich der Einschätzung ihrer eigenen Erziehungskompetenzen?
4. Zeigen sich je nach Niveau der elterlichen psychischen Belastung Unterschiede in der altersgerechten kindlichen Entwicklung und den entsprechenden Regulationsfähigkeiten?
5. In welchem Zusammenhang stehen die psychische Belastung der Eltern, die Selbsteinschätzung der Erziehungskompetenzen und die kindliche Entwicklung?

Die Studie „Kinder in Deutschland – KiD 0–3 2022“ wurde vom Bundesministerium für Familie, Senioren, Frauen und Jugend (BMFSFJ) im Rahmen der Bundesstiftung Frühe Hilfen aus dem Aktionsprogramm „Aufholen nach Corona für Kinder und Jugendliche“ der Bundesregierung gefördert.

## Methoden

### Studiendesign

Deutschlandweit wurden für die Studie 258 Kinder- und Jugendärztinnen bzw. -ärzte aus einer repräsentativen Adressdatei nach einem Zufallsprinzip ausgewählt. Eltern, die mit ihren Kindern zur Früherkennungsuntersuchung (U3 bis U7a) in die Praxen kamen, füllten einen Online-Erhebungsbogen u. a. zu ihren Belastungen und Ressourcen aus (Erhebungszeitraum: April bis Dezember 2022). Zusätzlich gaben die 258 teilnehmenden Ärztinnen und Ärzte anhand eines Dokumentationsbogens eine Einschätzung zur Gesundheit und Entwicklung des Kindes ab. Anonymität wurde für alle Beteiligten an der Studie gewährleistet, Datenschutzvorgaben wurden nachprüfbar eingehalten. Durchgeführt wurde die Erhebung von dem Forschungsinstitut House of Research, Berlin. Das Forschungsvorhaben erhielt ein positives Ethikvotum der Ethikkommission des Deutschen Jugendinstituts e. V. (Nr. 2021/005).

Insgesamt konnten Daten zu *N* = 7818 Familien mit Kindern im Alter von bis zu 3 Jahren erhoben werden. Zu 5591 Familien liegt sowohl der Elternfragebogen als auch der Arztfragebogen vor. Die Angaben der Eltern im Online-Fragebogen sowie die Angaben der Ärztinnen und Ärzte im ärztlichen Dokumentationsbogen wurden im Gesamtdatensatz fallbezogen zusammengeführt und auf Basis des Mikrozensus 2021 mittels einer Designgewichtung (Bundesland) und Poststratifizierungsgewichtung anhand der Variablen Bildung (kein Abschluss oder Hauptschule, polytechnische Oberschule 8.–9. Klasse/Realschulabschluss, polytechnische Oberschule 10. Klasse/(Fach‑)Hochschulreife), Staatsangehörigkeit (deutsch/nicht deutsch) und Familienform (alleinerziehend/nicht alleinerziehend) gewichtet [23].

Daten der Vorgängerstudie „KiD 0–3 2015“ [24] wurden von April bis September 2015 erhoben. Sie ermöglichen einen Vergleich der Prävalenzen psychischer Symptomatik der Jahre 2015 und 2022.

### Erhebungsinstrumente und Auswertung

#### Arztfragebogen.

Die Befunde zur Kindergesundheit basieren auf einem ärztlichen Dokumentationsbogen, den die Ärztinnen und Ärzte im Rahmen einer Früherkennungsuntersuchung ausfüllten. Dieses Erhebungsinstrument wurde in enger Kooperation mit dem Berufsverband der Kinder- und Jugendärzt*innen e. V. (BVKJ) entwickelt, einem Pretest unterzogen und vor der Durchführung der Studie von Ärztinnen und Ärzten des BVKJ im Praxisalltag erprobt.

Die Kinderärztinnen und -ärzte gaben an, ob bei dem Kind eine Grunderkrankung (d. h. eine chronische Erkrankung, körperliche oder geistige Behinderung, Entwicklungsstörung) vorliegt und ob sie Hinweise auf eine Regulationsstörung festgestellt haben (Antwortmöglichkeiten jeweils: Ja/Nein). Zudem wurden die Ärztinnen und Ärzte um eine Einschätzung der körperlichen, sozialen und affektiven Entwicklung des Kindes gebeten. Bewertet wurde die Entwicklung anhand der Kategorien „altersgerecht“, „teils nicht altersgerecht“ und „nicht altersgerecht“. Für die vorliegenden Analysen wurden die Angaben zur altersgerechten Entwicklung dichotomisiert, sodass Kinder, die insgesamt altersgerecht entwickelt sind, mit Kindern, die in mindestens einem Bereich (teils) nicht altersgerecht entwickelt sind, verglichen werden konnten.

#### Elternfragebogen.

Der Elternteil, der mit seinem Kind zur Früherkennung in die Arztpraxis kam, füllte einen Online-Fragebogen zu familialen Belastungen und Ressourcen aus. Die Fragen beziehen sich u. a. auf die Situation der Familie (bspw. die soziale Lage) und auf persönliche Merkmale des befragten Elternteils (bspw. seine psychische Belastung). Die Erhebungsinstrumente wurden vom Nationalen Zentrum Frühe Hilfen (NZFH) auf Basis einer umfangreichen Literaturrecherche entwickelt.

Die *psychische Belastung der Eltern* (hier: Anzeichen einer Depression oder Angstsymptomatik) wurde mittels der Kurzform des Patient Health Questionnaire (PHQ‑4; [25]) erhoben. Die 4 Items des PHQ‑4 erfassen Symptome von Depression (bspw. „Niedergeschlagenheit, Schwermut oder Hoffnungslosigkeit“) und generalisierter Angst (bspw. „nicht in der Lage sein, Sorgen zu stoppen oder zu kontrollieren“). Die Eltern gaben auf einer 4‑Punkt-Skala von 0 = „überhaupt nicht“ bis 3 = „jeden Tag“ an, wie häufig sie durch die genannten Symptome in den vergangenen 2 Wochen beeinträchtigt waren. Ab einem Cut-off von 6 liegt eine klinisch bedeutsame psychische Belastung vor. Ein Indexwert von 4 und 5 wird in dieser Studie als „moderate psychische Belastung“ unterhalb des klinisch relevanten Schwellenwertes verstanden (siehe auch [23]). Die interne Konsistenz in der vorliegenden Stichprobe lag bei Cronbachs α = 0,80.

Um die *soziale Lage (Armut)* einer Familie, die in Forschungsfrage 5 als Kontrollvariable genutzt wird, näherungsweise zu bestimmen, wurde in der zugrunde liegenden Studie ein konservativer, gut messbarer Armutsindikator herangezogen: der Bezug staatlicher Leistungen zur Grundsicherung (Arbeitslosengeld II; Sozialgeld nach Sozialgesetzbuch (SGB) II; Sozialhilfe; Bedarfsorientierte Grundsicherung) eines Haushaltsmitglieds in den letzten 12 Monaten.

Zur Erhebung der Selbsteinschätzung zu *Zweifeln an erzieherischen Kompetenzen* (bspw. „Einige Dinge in der Erziehung meines Kindes fallen mir schwerer, als ich erwartet hatte“) und *Schwierigkeiten im Einfühlungsvermögen* (bspw.: „Es fällt mir manchmal schwer herauszufinden, was mein Kind braucht“) wurden je 2 der 4 Items umfassenden Subskalen „Kompetenz“ und „Bindung“ aus der deutschen Fassung des Parenting Stress Index [26, 27] genutzt. Die Eltern machten Angaben auf einer Likert-Skala von 1 = „trifft gar nicht“ zu bis 5 = „trifft voll und ganz zu“. Die Items zu Zweifeln an der erzieherischen Kompetenz korrelierten in der vorliegenden Stichprobe miteinander mit *r* = 0,57, *p* < 0,001, die Items zur Einschätzung der Schwierigkeiten im Einfühlungsvermögen mit *r* = 0,54, *p* < 0,001. Die Einzelwerte wurden pro Skala summiert und dichotomisiert, sodass Gesamtwerte einer Befragungsperson von größer als 5,5 (skalierter Cut-off für 2 von 4 Items der Subskala) eine Belastung in dem jeweiligen Bereich indizierten [23].

Zur Erfassung der Selbsteinschätzung der *erzieherischen Selbstwirksamkeit* wurden die 5 Items der Subskala „Responsivität“ des Comprehensive Early Childhood Parenting Questionnaire verwendet (CECPAQ, [28]). Die Items spiegeln wider, wie selbstwirksam sich die Eltern im Umgang mit ihrem Kind empfinden (bspw. „Wenn mein Kind sich mit etwas schwertut, bin ich in der Lage, ihm zu helfen“). Die Eltern wählten auf einer Likert-Skala Antwortoptionen von 1 = „nie“ bis 6 = „immer“. Die interne Konsistenz war in der vorliegenden Stichprobe mit Cronbachs α = 0,78 zufriedenstellend. Die Einzelwerte wurden erst summiert und im nächsten Schritt dichotomisiert, sodass Werte ab 25 (1 Standardabweichung (SD) unter dem Mittelwert der Skala) eine geringe elterliche Selbstwirksamkeit der Befragungsperson indizieren [23].

#### Auswertungen.

Die Auswertungen erfolgten mit SPSS 26. Sie umfassten deskriptive Statistiken, Chi-Quadrat-Tests und logistische Regressionen. Für die Darstellung signifikanter Unterschiede wurde ein Grenzwert von *p* < 0,05 definiert. Vorhergehende Analysen der KiD-0–3-2022-Daten haben gezeigt, dass die Zusammenhänge zwischen familialen Belastungen und der kindlichen Entwicklung für Säuglinge deutlich geringer ausgeprägt waren als für Kleinkinder [29]. Aus diesem Grund wurden die Analysen zu Forschungsfrage 4 und 5 getrennt für Kinder unter einem Jahr und Kinder ab einem Jahr durchgeführt.

## Ergebnisse

Die große Mehrheit der teilnehmenden Elternteile waren Mütter (91,6 %). Die Kinder waren im Durchschnitt 15,2 Monate alt (SD: 0,16). 10,3 % der Eltern gaben an, dass sie selbst oder Mitglieder ihres Haushalts in den vergangenen 12 Monaten staatliche Leistungen zur Grundsicherung erhalten hatten. Insgesamt waren 5,9 % der teilnehmenden Eltern alleinerziehend. Im Folgenden werden die Ergebnisse zu den Forschungsfragen berichtet.

### 1. Wie weit verbreitet sind psychische Belastungen (bzw. Symptome einer Depression oder einer Angststörung) bei Eltern von kleinen Kindern?

Von den befragten Eltern berichteten 5,9 % (6,1 % der Mütter und 3,5 % der Väter, χ^2^ (df = 1) = 4,88, *p* < 0,05) klinisch bedeutsame Symptome einer Depression oder Angststörung (PHQ-4-Summenscore ≤ 6). Weitere 15,6 % der Befragten berichteten Symptome, die auf eine moderate psychische Belastung hinweisen (PHQ-4-Wert 4 oder 5). Insgesamt war damit im Jahr 2022 in Deutschland etwa ein Fünftel (21,5 %) aller Mütter und Väter, die ihr Kind zur U3 bis U7a begleiteten, psychisch belastet.

### 2. Ist der Anteil psychisch belasteter Eltern in den letzten Jahren angestiegen?

**Abb. 1:**
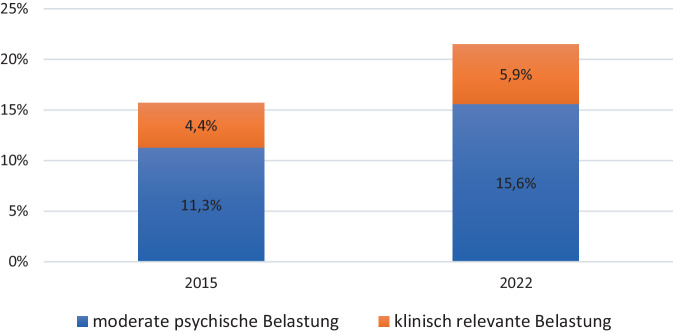
Elterliche psychische Belastung in den Jahren 2015 und 2022. Angaben von *N* = 5591 Familien, Daten sind gewichtet mittels einer Designgewichtung (Bundesland) und Poststratifizierungsgewichtung (Bildung, Staatsangehörigkeit, alleinerziehend). Eigene Abbildung

Die Vorgängerstudie aus dem Jahr 2015 [24] ergab bei den befragten Eltern Prävalenzen einer psychischen Belastung von insgesamt 15,7 % (eigene Berechnungen). 4,4 % der Eltern berichteten 2015 eine Symptomatik, die als klinisch relevant definiert wird, weitere 11,3 % waren moderat psychisch belastet (Abb. 1). Somit zeigten sich sowohl für die moderate psychische Belastung (prozentuale Veränderung [(Endwert-Anfangswert)/Endwert × 100]: +38 %) als auch für die klinisch bedeutsame psychische Symptomatik (prozentuale Veränderung: +34 %) im Jahr 2022 höhere Prävalenzen als noch 2015.

### 3. Wie unterscheiden sich Eltern mit unterschiedlichen psychischen Belastungsniveaus hinsichtlich der Einschätzung ihrer eigenen Erziehungskompetenzen?

**Abb. 2:**
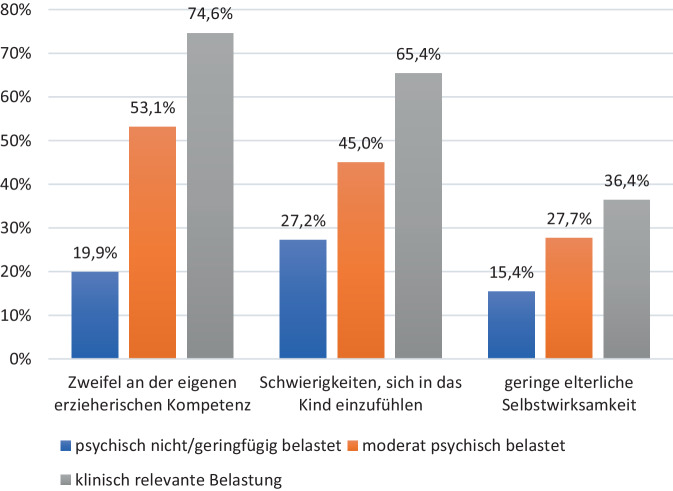
Elterliche psychische Belastung und subjektive Erziehungskompetenz. Angaben von *N* = 5591 Familien, Daten sind gewichtet mittels einer Designgewichtung (Bundesland) und Poststratifizierungsgewichtung (Bildung, Staatsangehörigkeit, alleinerziehend). Eigene Abbildung

Um die Frage zu beantworten, wurden 3 Gruppen miteinander verglichen: Eltern ohne oder mit nur geringfügiger psychischer Belastung, psychisch moderat belastete Eltern und Eltern mit klinisch bedeutsamer psychischer Symptomatik. Es zeigten sich statistisch signifikante Gruppenunterschiede in der Selbsteinschätzung bei allen untersuchten Konstrukten (Abb. 2). 74,6 % der klinisch bedeutsam psychisch belasteten Eltern äußerten Zweifel an ihren elterlichen Kompetenzen (gegenüber bspw. nur 19,9 % der Eltern, die keine oder eine geringfügige psychische Belastung angaben, χ^2^(df = 2) = 731,96, *p* < 0,001). 65,4 % der Eltern mit klinisch bedeutsamer Symptomatik berichteten von Schwierigkeiten, sich in ihr Kind einzufühlen. Bei den Eltern ohne oder mit geringfügiger psychischer Belastung waren dies mit 27,2 % deutlich weniger, χ^2^(df = 2) = 270,49, *p* < 0,001. Stark ausgeprägte Gruppenunterschiede zeigten sich auch hinsichtlich der Selbstwirksamkeit der Eltern im Umgang mit ihrem Kind, χ^2^(df = 2) = 139,93, *p* < 0,001.

### 4. Zeigen sich je nach Niveau der elterlichen psychischen Belastung Unterschiede in der altersgerechten kindlichen Entwicklung und den entsprechenden Regulationsfähigkeiten?

Im Folgenden wird untersucht, wie sich der Entwicklungsstand der Kinder mit einem Elternteil, das Symptome einer klinisch relevanten psychischen Belastung berichtete, von Kindern mit Eltern ohne klinisch bedeutsame Symptomatik unterscheidet.

Ärztinnen und Ärzte dokumentierten bei Säuglingen, die von einem Elternteil mit klinisch bedeutsamen psychischen Belastungen begleitet wurden, häufiger Hinweise auf eine Regulationsstörung als bei Säuglingen ohne klinisch bedeutsam belasteten Elternteil (15,9 % versus 8,7 % Chi-Quadrat (df = 1) = 7,89, *p* < 0,01). Bei der ärztlichen Einschätzung, inwieweit die Entwicklung altersgerecht ist, zeigten sich für Kinder unter einem Jahr keine statistisch signifikanten Unterschiede.

Bei Kleinkindern dokumentierten die Ärztinnen und Ärzte häufiger Hinweise auf Regulationsstörungen für Kinder mit Elternteil mit klinisch bedeutsamer psychischer Belastung als für Kinder ohne einen derart belasteten Elternteil (14,8 % versus 9,4 %, Chi-Quadrat (df = 1) = 5,79, *p* < 0,05). Bei der ärztlichen Beurteilung, inwieweit die Entwicklung altersgerecht ist, zeigten sich deutliche und statistisch signifikante Unterschiede: Eine zumindest teils nicht altersgerechte Entwicklung stellten die Ärztinnen und Ärzte bei 26,7 % der Kinder fest, die von einem Elternteil, der eine klinisch relevante psychische Symptomatik berichtete, in die Arztpraxis begleitet wurden. Bei allen anderen Kindern waren dies nur 15,7 % (χ^2^(df = 1) = 14,90, *p* < 0,001).

### 5. In welchem Zusammenhang stehen die psychische Belastung der Eltern, die Selbsteinschätzung der Erziehungskompetenzen und die kindliche Entwicklung?

Um ein genaueres Bild von den Zusammenhängen zu erhalten zwischen a) einer klinisch bedeutsamen psychischen Symptomatik bei dem Elternteil, der sein Kind zur Früherkennungsuntersuchung begleitete, b) den selbst eingeschätzten Erziehungskompetenzen und c) der ärztlich dokumentierten kindlichen Entwicklung, wurden multiple logistische Regressionen durchgeführt. Da eine Grunderkrankung des Kindes sowohl zu einer Entwicklungsverzögerung als auch zu einer erhöhten psychischen Belastung der Eltern beitragen kann [30], wurde das Vorliegen einer Grunderkrankung in die Regressionsmodelle mit aufgenommen. Auch die Variable Armut wurde in den Modellen berücksichtigt, da vorherige Analysen der KiD-0–3-2022-Daten einen Zusammenhang zwischen einer familialen Armutslage und der kindlichen Entwicklung gezeigt haben [29]. Für die Variablen „Zweifel an der elterlichen Kompetenz“, „Schwierigkeiten im Einfühlungsvermögen“ und „geringe elterliche Selbstwirksamkeit“ wurden jeweils einzelne Regressionsmodelle berechnet.

### Modelle zur Vorhersage von Hinweisen auf eine Regulationsstörung

Da die psychische Belastung eines Elternteils sowohl für Säuglinge als auch für Kleinkinder mit ärztlich dokumentierten Hinweisen auf Regulationsstörungen assoziiert war, wurde für die Regressionsmodelle zur statistischen Vorhersage von Regulationsstörungen die gesamte Stichprobe (Säuglinge und Kleinkinder) berücksichtigt. Im ersten Modell (1a) zeigt sich, dass die psychische Belastung des Elternteils auch dann mit einer Regulationsstörung bei Säuglingen und Kleinkindern zusammenhängt, wenn für eine Grunderkrankung des Kindes und für Armut der Familie kontrolliert wird (wobei Armut keinen statistisch signifikanten Zusammenhang mit Hinweisen auf eine Regulationsstörung zeigt). Die Modelle 2a–4a zeigen Zusammenhänge zwischen der elterlichen psychischen Belastung und der elterlichen Selbsteinschätzung ihrer Erziehungskompetenzen auf der einen Seite und Hinweisen auf Regulationsstörungen auf der anderen Seite. Berichteten die Eltern von Zweifeln an der elterlichen Kompetenz (Modell 2a), Schwierigkeiten im Einfühlungsvermögen (Modell 3a) oder einer geringen elterlichen Selbstwirksamkeit (Modell 4a) erhöhte sich die Wahrscheinlichkeit von Hinweisen auf eine Regulationsstörung jeweils um das 1,9- bis 2,1-Fache. Durch das Hinzufügen der Variable „Zweifel an der eigenen erzieherischen Kompetenz“ verliert der Zusammenhang zwischen der elterlichen psychischen Belastung und den Hinweisen auf eine Regulationsstörung seine statistische Signifikanz (Modell 2a), sodass hier von einem vermittelnden Effekt ausgegangen werden kann. Die vergleichsweise niedrigen Werte für Nagelkerkes R^2^ machen aber auch deutlich, dass Hinweise auf Regulationsstörungen mit den hier betrachteten Variablen nur zu einem geringen Teil erklärt werden können.

### Modelle zur Vorhersage der altersgerechten Entwicklung von Kleinkindern

Regressionsmodelle zur statistischen Vorhersage einer nichtaltersgerechten Entwicklung wurden auf die Substichprobe der Kleinkinder bezogen, da für Säuglinge keine signifikanten Unterschiede in der Häufigkeit einer nichtaltersgerechten Entwicklung zwischen Kindern mit und ohne einen klinisch bedeutsam psychisch belasteten Elternteil gefunden wurden. Modell 1b zeigt, dass eine klinisch bedeutsame psychische Belastung auch dann negativ mit der altersgerechten Entwicklung assoziiert ist, wenn für das Vorliegen einer Grunderkrankung des Kindes und für Armut der Familie kontrolliert wird. Alle Modelle waren statistisch signifikant (χ^2^; Tab. 1). Die Modelle unter Einschluss der Erziehungskompetenz erklärten jeweils 13,0 % der Varianz einer altersgerechten Entwicklung. Kleinkinder mit einer Grunderkrankung hatten eine 5,6-fach erhöhte Wahrscheinlichkeit einer (teils) nichtaltersgerechten Entwicklung und Kinder aus Familien in Armut eine etwa doppelt so hohe. Auch unter Berücksichtigung dieser bedeutsamen Prädiktorvariablen für eine nichtaltersgerechte Entwicklung hatten Kleinkinder aus Familien mit einem Elternteil, der klinisch bedeutsame Symptome einer psychischen Belastung berichtete, gegenüber allen anderen Kleinkindern eine mindestens 1,6-fach erhöhte Wahrscheinlichkeit für eine (teils) nichtaltersgerechte Entwicklung. Darüber hinaus hatte auch die Selbsteinschätzung der Erziehungskompetenzen eine eigenständige Erklärungskraft. Berichteten die Eltern von Zweifeln an der elterlichen Kompetenz (Modell 2b), Schwierigkeiten im Einfühlungsvermögen (Modell 3b) oder einer geringen elterlichen Selbstwirksamkeit (Modell 4b), erhöhte sich die Wahrscheinlichkeit einer nichtaltersgerechten Entwicklung um jeweils das 1,3- bis 1,4-Fache.

**Tab. 1:** Ergebnisse logistische Regressionsmodelle zur statistischen Vorhersage von Hinweisen auf Regulationsstörungen (für alle Kinder) und altersgerechter Entwicklung (für Kinder ab dem ersten Lebensjahr). Angaben von *N* = 5591 Familien (in Prozent), Daten sind gewichtet mittels einer Designgewichtung (Bundesland) und Poststratifizierungsgewichtung (Bildung, Staatsangehörigkeit, alleinerziehend)

		a) Hinweise auf eine Regulationsstörung		b) Nichtaltersgerechte Entwicklung	
**Modelle im Vergleich**	Prädiktorvariablen	Odds Ratio	*p*	Odds Ratio	*p*
**Modell 1a** Χ^2^(df = 3) = 37,83; *p* < 0,001; Nagelkerkes R^2^ = 0,02 **Modell 1b** Χ^2^(df = 3) = 209,72; *p* < 0,001; Nagelkerkes R^2^ = 0,12	Grunderkrankung	1,90	< 0,001	5,64	< 0,001
	Armut	1,22	n. s.	2,06	< 0,001
	Psychische Belastung	1,84	< 0,001	1,85	< 0,01
**Modell 2a** Χ^2^(df = 4) = 87,89; *p* < 0,001; Nagelkerkes R^2^ = 0,04 **Modell 2b** Χ^2^(df = 4) = 214,16; *p* < 0,001; Nagelkerkes R^2^ = 0,13	Grunderkrankung	1,82	< 0,001	5,60	< 0,001
	Armut	1,21	n. s.	2,06	< 0,001
	Psychische Belastung	1,27	n. s.	1,61	< 0,05
	Zweifel an der elterlichen Kompetenz	2,08	< 0,001	1,30	< 0,01
**Modell 3a** Χ^2^(df = 4) = 89,86; *p* < 0,001; Nagelkerkes R^2^ = 0,04 **Modell 3b** Χ^2^(df = 4) = 217,41; *p* < 0,001; Nagelkerkes R^2^ = 0,13	Grunderkrankung	1,86	< 0,001	5,62	< 0,001
	Armut	1,21	n. s.	2,05	< 0,001
	Psychische Belastung	1,46	< 0,05	1,68	< 0,01
	Schwierigkeiten im Einfühlungsvermögen	2,02	< 0,001	1,35	< 0,05
**Modell 4a** Χ^2^(df = 4) = 66,76; *p* < 0,001; Nagelkerkes R^2^ = 0,03 **Modell 4b** Χ^2^(df = 4) = 216,02; *p* < 0,001; Nagelkerkes R^2^ = 0,13	Grunderkrankung	1,85	< 0,001	5,55	< 0,001
	Armut	1,19	n. s.	2,06	< 0,001
	Psychische Belastung	1,62	< 0,01	1,73	< 0,01
	Geringe elterliche Selbstwirksamkeit	1,83	< 0,001	1,37	< 0,05

*n.* *s.* nicht signifikant

Der Χ^2^-Test für das Modell gibt an, ob das Modell insgesamt signifikant ist oder nicht

*p* = Signifikanzniveau

Nagelkerkes R^2^ = Varianzaufklärung der logistischen Regression

## Diskussion

Zusammenfassend zeigen die Ergebnisse der bundesweiten Repräsentativbefragung KiD 0–3 aus dem Jahr 2022, dass ein Fünftel der Eltern kleiner Kinder psychisch belastet ist. Damit hat sich der Anteil psychisch belasteter Eltern seit 2015 erhöht. Auch wenn insgesamt mehr Eltern von psychischen Belastungen berichten, bestehen weiterhin deutliche Zusammenhänge mit frühkindlichen Entwicklungsauffälligkeiten. Dabei spielen Einschränkungen der eigenen Erziehungskompetenzen bzw. eine negative Einschätzung dieser Kompetenzen eine Rolle.

Im Jahr 2022 berichteten knapp 6 % der Eltern, die ihre Kinder zur Früherkennung in die kinderärztliche Praxis begleiteten (U3 bis U7a), von Symptomen einer Depressions- oder Angststörung, die auf eine klinisch relevante psychische Belastung schließen lassen. Weitere 15,6 % der befragten Eltern berichteten moderat ausgeprägte Symptome. Gegenüber der Studie KiD 0–3 aus dem Jahr 2015 zeigt sich somit ein Anstieg sowohl bei der klinisch relevanten psychischen Belastung (2015: 4,4 %) als auch bei der moderaten psychischen Belastung (2015: 11,3 %). Diese Befunde stimmen mit Statistiken zur Zunahme psychischer Diagnosen in Deutschland überein [31] und können eine Folge gestiegener Herausforderungen in den letzten Jahren sein: neben Kriegsgefahren sowie Klima- und Energiekrisen insbesondere auch die COVID-19-Pandemie. So gab in weiteren Analysen des KiD-0–3-2022-Datensatzes ein Viertel der befragten Eltern an, dass die Zeit der Pandemie sie persönlich oder ihre Familie stark belastet habe [20]. Durch die COVID-19-Pandemie hat das Thema psychische Gesundheit in der Öffentlichkeit zudem an Bedeutung gewonnen, sodass bei einigen Eltern sowohl die Sensibilität für Symptome einer Depression oder Angststörung gestiegen als auch die Hemmschwelle, diese zu berichten, gesunken sein könnte [31]. Zukünftige Studien werden zeigen, ob es sich bei dem hier festgestellten Anstieg der berichteten psychischen Belastungen tatsächlich um einen Anstieg aufgrund der beschriebenen Krisen handelt oder ob er einen langfristigen Trend anzeigt, der auf eine zunehmende Belastung von Eltern kleiner Kinder bzw. eine zunehmende Bereitschaft, Belastungen zu berichten, hindeutet. Bei der Interpretation der Befunde zur Verbreitung psychischer Belastungen von Eltern ist auch zu bedenken, dass in der vorliegenden Studie nur wenige Familien mit unmittelbarer Einwanderungs- und Fluchterfahrung teilgenommen haben (indiziert über geringe deutsche Sprachkenntnisse; [32]), obwohl der Anteil dieser Familien seit 2015 stark gewachsen ist. Da gerade Familien mit Fluchterfahrungen häufig traumatisierende Erfahrungen gemacht haben (siehe auch den Beitrag von Chakraverty et al. in diesem Themenheft) oder mit den Folgen von Deprivation im Herkunftsland und unter schwierigen Bedingungen in Deutschland leben [33], ist davon auszugehen, dass sie auch häufiger psychisch belastet sind. Der tatsächliche Anteil von Müttern und Vätern mit klinisch bedeutsamer psychischer Belastung dürfte also noch höher sein.

Die vorliegenden Ergebnisse zeigen, dass Eltern mit klinisch bedeutsamen Symptomen einer psychischen Belastung ihre eigenen Erziehungskompetenzen als deutlich geringer einschätzen als Eltern ohne oder mit weniger stark ausgeprägter psychischer Belastung. So zweifeln Eltern, die eine klinisch bedeutsame Symptomatik berichten, im Vergleich zu den anderen Eltern etwa 3‑mal so häufig an ihren erzieherischen Kompetenzen. Geringere Erziehungskompetenzen oder ein dysfunktionaleres Erziehungsverhalten, etwa bei depressiven Eltern, werden von vielen anderen Studien bereits belegt [10–13]. Des Weiteren stimmen die hier belegten Zusammenhänge mit den schon früh postulierten [17] und später auch durch neurobiologische Befunde unterstützten [34] Zusammenhängen zwischen Depression und Angststörungen und deren Einfluss auf negative Kognitionen überein. Negative Kognitionen umfassen neben der negativen Sicht der Welt und der Zukunft auch eine negative Sicht auf das Selbst. Auch für Kognitionen, die das Elternsein betreffen, wurden in anderen Studien Verzerrungen durch Angststörungen und Depressionen berichtet [4].

In der ärztlichen Dokumentation zeigt sich, dass die psychische Belastung der Eltern mit der Regulationsfähigkeit der Kinder zusammenhängt. Im Kleinkindalter ist sie zusätzlich mit einer nichtaltersgerechten Entwicklung assoziiert. Bemerkenswert ist, dass dies auch dann der Fall ist, wenn für eine Grunderkrankung des Kindes und die soziale Lage der Familie, beides relevante Prädiktoren für Entwicklungsrisiken, kontrolliert wurde. Weiterführende Regressionsanalysen zeigten direkte (und indirekte) Zusammenhänge zwischen der elterlichen psychischen Belastung und der kindlichen Entwicklung, zum Teil vermittelt über die Einschätzung der eigenen Erziehungskompetenzen. Diese Befunde stimmen mit Erkenntnissen aus anderen Studien überein, die Zusammenhänge elterlicher Psychopathologie und einer beeinträchtigten sozioemotionalen Entwicklung von Kindern belegen [8], zum Teil mediiert über Belastungen in der Elternrolle [35]. Der Befund, dass auch bei einer offenbar zunehmenden Verbreitung psychischer Belastungen in der Elternpopulation derart deutliche Zusammenhänge mit elterlichen Erziehungskompetenzen und frühen Entwicklungsauffälligkeiten gefunden werden, legt nahe, dass der beobachtete Anstieg psychischer Belastungen nicht ausschließlich auf eine gestiegene Sensibilität gegenüber psychischen Belastungen oder eine gesunkene Hemmschwelle, Symptome zu berichten, zurückzuführen ist. Wäre der hypothetisierte Anstieg nur auf eine in der Bevölkerung gestiegene Sensibilität und eine geringere Schwelle, psychische Belastungen zu berichten, zurückzuführen ohne einen dahinterstehenden tatsächlichen Anstieg der Symptome, sollte der Anstieg nur in geringem Maße mit Hinweisen auf kindliche Regulationsstörungen und Entwicklungsverzögerungen assoziiert sein.

Diese Studie hat neben Stärken auch einige Limitationen, die bei der Interpretation der Daten berücksichtigt werden müssen. Mit der Repräsentativerhebung KiD 0–3 wurden bundesweite Verteilungsdaten zu einer Vielzahl von Themen generiert. Um die Studienteilnehmenden nicht zu überlasten und damit ein oberflächliches Ausfüllen oder sogar einen Abbruch zu riskieren, konnten deshalb zu jedem Thema nur Einzelitems oder kurze (Sub)Skalen in das Befragungskonzept integriert werden. Zudem können bei Selbstberichten zur psychischen Gesundheit Effekte sozialer Erwünschtheit oder andere Verzerrungen nicht ausgeschlossen werden. Des Weiteren konnte zwar eine große Stichprobe, insbesondere Mütter, in unterschiedlichen Lebenslagen durch die repräsentativ angelegte Befragung erreicht werden. Allerdings konnte jeweils nur ein Elternteil befragt werden. Auch wenn der Elternteil, der seinen Säugling oder sein Kleinkind zu den Früherkennungsuntersuchungen in die Arztpraxis begleitet, vermutlich eine zentrale Bezugsperson für das Kind ist, können psychische Belastungen des anderen Elternteils (oder weiterer wichtiger Bezugspersonen) ebenso bedeutsam sein. Beispielsweise ist das Risiko einer Übertragung elterlicher Depression auf die Kinder etwa doppelt so hoch, wenn beide Elternteile betroffen sind [36]. Da mehrheitlich Mütter an der Befragung teilgenommen haben, können keine Aussagen zu etwaigen Unterschieden zwischen Müttern und Vätern getroffen werden. Darüber hinaus ist zu beachten, dass es sich bei dem Konzept der Regulationsstörungen, gerade im Kontext der Diagnostik während der Vorsorgeuntersuchungen, um ein sehr subjektives Konstrukt handelt. Unterschiedliches Vorgehen in der Diagnosestellung kann zu unterschiedlichen Bewertungen führen. Aufgrund des Querschnittcharakters der Studie kann letztlich nicht über die Richtung der Effekte entschieden werden. So legen Längsschnittstudien bspw. den Schluss nahe, dass frühkindliche Regulationsstörungen auch zu einem beeinträchtigten psychischen Wohlbefinden der Eltern führen und prädiktiv für eine spätere Depression oder Angstsymptomatik der Eltern sein können [37, 38].

## Fazit

Die Ergebnisse der vorliegenden Studie zu Zusammenhängen zwischen der elterlichen psychischen Belastung, der elterlichen Einschätzung ihrer Erziehungskompetenzen und der Entwicklung von Säuglingen und Kleinkindern legen eindrücklich nahe, dass psychisch belastete Eltern von Unterstützung bei der Ausübung ihrer Elternrolle zum Wohl ihrer Kinder profitieren könnten. Die Ergebnisse bekräftigen damit die Empfehlungen der Interministeriellen Arbeitsgruppe „Gesundheitliche Auswirkungen auf Kinder und Jugendliche durch Corona“ [39] zu den Frühen Hilfen. Diese beschreiben die Unterstützung der Familien bei der Entwicklung von Elternkompetenzen, den Abbau von Unsicherheiten in der Elternrolle sowie den Aufbau einer entwicklungsförderlichen Eltern-Kind-Beziehung als zentrale Aufgaben der Fachkräfte in den Frühen Hilfen. Damit psychisch belastete Eltern und ihre Kinder von den Frühen Hilfen profitieren können, ist die interprofessionelle Kooperation zwischen Fachkräften, die junge Familien begleiten und unterstützen, von entscheidender Bedeutung. Fachkräfte in den Frühen Hilfen und deren Kooperationspartner in den Netzwerken Frühe Hilfen (z. B. Kinderärztinnen und -ärzte) können psychische Belastungen bei den Eltern erkennen, sie angemessen thematisieren und Eltern dazu motivieren, therapeutische Hilfe in Anspruch zu nehmen. Umgekehrt können Fachkräfte in der ambulanten und stationären Psychiatrie und Psychotherapie bei Patientinnen und Patienten mit kleinen Kindern auch deren Elternkompetenzen in den Blick nehmen, um bei Bedarf weitere Unterstützungsangebote, z. B. die Begleitung durch eine Familienhebamme, zu vermitteln. Durch die hier gezeigte Zunahme der elterlichen psychischen Belastung seit 2015 wird deutlich, wie wichtig es ist, die psychische Belastung von Eltern systemübergreifend in den Blick zu nehmen.
